# KPNB1 Inhibitor Importazole Reduces Ionizing Radiation-Increased Cell Surface PD-L1 Expression by Modulating Expression and Nuclear Import of IRF1

**DOI:** 10.3390/cimb43010013

**Published:** 2021-05-19

**Authors:** Hironori Yoshino, Yoshiaki Sato, Manabu Nakano

**Affiliations:** 1 Department of Radiation Science, Hirosaki University Graduate School of Health Sciences, Hirosaki, Aomori 036-8564, Japan; h20gg702@hirosaki-u.ac.jp; 2 Department of Bioscience and Laboratory Medicine, Hirosaki University Graduate School of Health Sciences, Hirosaki, Aomori 036-8564, Japan; mnakano@hirosaki-u.ac.jp

**Keywords:** karyopherin-β1, ionizing radiation, programmed death-ligand 1, interferon regulatory factor 1

## Abstract

Programmed death-ligand 1 (PD-L1) is an immune checkpoint molecule that negatively regulates anti-tumor immunity. Recent reports indicate that anti-cancer treatments, such as radiation therapy, increase PD-L1 expression on the surface of tumor cells. We previously reported that the nuclear transport receptor karyopherin-β1 (KPNB1) is involved in radiation-increased PD-L1 expression on head-and-neck squamous cell carcinoma cells. However, the mechanisms underlying KPNB1-mediated, radiation-increased PD-L1 expression remain unknown. Thus, the mechanisms of radiation-increased, KPNB1-mediated PD-L1 expression were investigated by focusing on the transcription factor interferon regulatory factor 1 (IRF1), which is reported to regulate PD-L1 expression. Western blot analysis showed that radiation increased IRF1 expression. In addition, flow cytometry showed that IRF1 knockdown decreased cell surface PD-L1 expression of irradiated cells but had a limited effect on non-irradiated cells. These findings suggest that the upregulation of IRF1 after irradiation is required for radiation-increased PD-L1 expression. Notably, immunofluorescence and western blot analyses revealed that KPNB1 inhibitor importazole not only diffused nuclear localization of IRF1 but also decreased IRF1 upregulation by irradiation, which attenuated radiation-increased PD-L1 expression. Taken together, these findings suggest that KPNB1 mediates radiation-increased cell surface PD-L1 expression through both upregulation and nuclear import of IRF1.

## 1. Introduction

Programmed death-ligand 1 (PD-L1/CD274) is an immune checkpoint molecule expressed on the surface of immune cells, such as dendritic cells and macrophages [1]. The interaction of PD-L1 with its receptor, programmed death-1 (PD-1), causes immune suppression through the inhibition of effector T cell function [1]. PD-L1, which is also expressed on various types of cancer cells, is associated with clinical outcomes [2]. For example, in some cancers, such as oral squamous cell carcinoma and lung cancers, high expression of PD-L1 has been correlated with poor prognosis and malignancy [3,4,5]. Therefore, regulation of PD-L1 expression could improve the efficacy of cancer therapies. Importantly, recent studies have demonstrated that various treatments, including radiation therapy and chemotherapy, increase PD-L1 expression against various cancers [6,7]. However, the mechanisms underlying increased PD-L1 expression on cancer cells remain unclear.

Karyopherin-α (KPNA) and karyopherin-β1 (KPNB1) are nuclear transport receptors, and selectively aid the shuttle of karyophilic proteins harboring nuclear localization signals (NLSs) through the nuclear pore complex [8,9]. In brief, KPNA recognizes and binds to NLS-containing cargo, while KPNB1 mediates the docking of KPNA/NLS-containing cargo to the nuclear pore complex, thereby facilitating the nuclear entry of the target cargo. Seven subtypes of human KPNA with cargo specificity have been identified to date [10].

Recent evidence indicates the involvement of members of the karyopherin family in the progression of various cancers [11,12], indicating that these molecules are promising targets for cancer treatment. Our group recently reported that KPNB1 is involved in the malignancy of head-and-neck squamous cell carcinoma (HNSCC) [13]. In addition, a previous study demonstrated that KPNB1 regulates the radioresistance of HNSCC cells. Of note, KPNB1 inhibition significantly attenuated radiation-increased PD-L1 expression on the surface of HNSCC cells [13]. These results suggest that KPNB1 is a promising target to improve the efficacy of radiation therapy against HNSCC. However, the mechanisms underlying KPNB1-mediated, radiation-increased PD-L1 expression remain unknown.

Interferon regulatory factor 1 (IRF1) is a transcription factor and plays an important role in the immune response, such as the induction of anti-viral cytokine interferons [14]. It is reported that IRF1 contains an NLS [15] and is transported into the nucleus by KPNA2 [16]. Recent studies showed that IRF1 is involved in not only interferon-γ-induced PD-L1 expression but also DNA double-strand break (DSB)-induced PD-L1 upregulation [7,17]. Furthermore, in silico analysis of cohorts included in The Cancer Genome Atlas (TCGA) in a previous study by our group showed that the transcripts of PD-L1 were significantly correlated to that of IRF1 levels in various cancers, including HNSCC [13], suggesting that KPNB1 regulates PD-L1 expression on the surface of irradiated cells via IRF1 regulation. Therefore, the aim of the present study was to investigate the involvement of IRF1 in radiation-increased, KPNB1-mediated cell surface PD-L1 expression. The findings of this study suggest that the upregulation and nuclear import of IRF1 are required for KPNB1-mediated, radiation-increased, cell-surface PD-L1 expression.

## 2. Materials and Methods

### 2.1. Reagents

The KPNB1 inhibitor importazole (IPZ) and dimethyl sulfoxide (DMSO) were purchased from Sigma-Aldrich (St. Louis, MO, USA). β-actin antibody (#4967), GAPDH (D16H11) XP^®^ rabbit monoclonal antibody (#5174), IRF1 (D5E4) XP^®^ rabbit monoclonal antibody (#8478), KPNA2 rabbit antibody (#14372), and anti-rabbit IgG horseradish peroxidase (HRP)-conjugated antibody (#7074) were purchased from Cell Signaling Technology Japan, K.K. (Tokyo, Japan). Phycoerythrin (PE)-labeled anti-human PD-L1 monoclonal antibody (#329706) and PE-conjugated anti-mouse IgG_2b_ antibody (#400314) were purchased from BioLegend (San Diego, CA, USA). Ambion Silencer^®^ Select Pre-designed small interfering RNA (siRNA) against the genes encoding IRF1 (cat. no. s7501) and KPNA2 (cat. no. s7922), and Silencer^®^ Select Negative #1 Control siRNA (cat. no. AM4611) were purchased from Thermo Fisher Scientific (Waltham, MA, USA).

### 2.2. Cell Culture and Treatment

Human HNSCC Ca9-22 cells and human lung adenocarcinoma (LUAD) A549 cells were obtained from the RIKEN Bio-Resource Research Center (Tsukuba, Japan). Ca9-22 cells were maintained in high-glucose Dulbecco’s modified Eagle’s medium (DMEM; Wako Pure Chemical Industries, Ltd., Osaka, Japan) supplemented with 10% heat-inactivated fetal bovine serum (FBS; Sigma-Aldrich) and 1% penicillin/streptomycin (P/S; Wako Pure Chemical Industries). A549 cells were maintained in low-glucose DMEM (Sigma-Aldrich) supplemented with 1% P/S and 10% FBS. Both cell lines were cultured at 37 °C under a humidified atmosphere of 5% CO_2_/95% air.

Ca9-22 cells (10 × 10^4^) and A549 cells (8 × 10^4^) were seeded in 35 mm culture dishes (Sumitomo Bakelite Co., Ltd., Tokyo, Japan) and incubated for 6 h to allow adherence to the dishes. After incubation, the cells were treated with 10 μM IPZ or X-ray irradiation. DMSO-treated cells were prepared as vehicle control for IPZ. In some experiments, X-ray irradiation was performed at 1 h after IPZ administration. The cells were cultured for the indicated time periods at 37 °C and then harvested for subsequent analysis.

### 2.3. In Vitro X-ray Irradiation

X-ray irradiation (150 kVp, 20 mA, 0.5 mm Al, and 0.3-mm Cu filters) was performed using an X-ray generator (MBR-1520R-3; Hitachi Medical Corporation, Tokyo, Japan) at a distance of 45 cm from the focus and a dose rate of 0.99–1.03 Gy/min.

### 2.4. siRNA Transfection

The cells were transfected with siRNA-targeting IRF1 or KPNA2, or control siRNA using Lipofectamine^®^ RNAiMAX reagent (Invitrogen; Thermo Fisher Scientific, Inc., Waltham, MA, USA) in accordance with the manufacturer’s protocol, at a final siRNA concentration of 10 nM. After transfection for 48 h, the cells were harvested for subsequent analyses.

### 2.5. Analysis of Cell Surface PD-L1 Expression

The irradiated cells were cultured for 4 days and harvested for analysis of cell surface PD-L1 expression as previously reported [13]. In brief, harvested cells were washed twice with phosphate-buffered saline (PBS) and then stained with PE-conjugated anti-human PD-L1 antibody or a PE-conjugated anti-mouse IgG_2b_ isotype control for 30 min at 4 °C in the dark. After staining, the cells were washed and analyzed using flow cytometry (Cytomics FC500; Beckman Coulter, Inc., Brea, CA, USA).

### 2.6. Sodium Dodecyl Sulfate Polyacrylamide Gel Electrophoresis (SDS-PAGE) and Western Blot Analysis

SDS-PAGE and western blot analysis were performed as previously reported [18]. Western blot analysis was performed with antibodies against IRF1 (dilution, 1:3000), KPNA2 (1:3000), GAPDH (1:4000), and β-actin (1:4000). HRP-conjugated anti-rabbit IgG antibody (1:10,000) was used as a secondary antibody. The antigens were visualized using Clarity^TM^ Western ECL Substrate (Bio-Rad Laboratories, Inc., Hercules, CA, USA). The blots were stripped with Stripping Solution (Wako Pure Chemical Industries, Ltd.). Quantification of the bands was performed using ImageJ software (National Institutes of Health, Bethesda, MD, USA).

### 2.7. Immunofluorescence Analysis

Cells were seeded onto glass slides (5712-002; IWAKI^®^; AGC Techno Glass Co., Ltd.), incubated for 6 h, and then treated with IPZ and/or X-ray irradiation. The treated cells were fixed for 15 min with 4% paraformaldehyde in Ca^2+^ and Mg^2+^-free PBS and then blocked with blocking buffer (3% bovine serum albumin (BSA)/0.3% Triton X-100 in PBS) for 60 min at room temperature. The coverslips were then incubated overnight with IRF1 primary antibody diluted to 1:300 with antibody dilution buffer (1% BSA/0.3% Triton X-100 in PBS). After incubation, the samples were washed 3 times and incubated with AlexaFluor 488^®^-conjugated anti-rabbit IgG secondary antibody (#4412; Cell Signaling Technology Japan, K.K.) diluted to 1:400 with antibody dilution buffer for 1 h at room temperature in the dark. After washing 3 times, the samples were mounted using Vectashield^®^ Mounting Medium with 4′,6-diamidino-2-phenylindole (Vector Laboratories, Inc., Burlingame, CA, USA) and examined under an LSM 710 laser-scanning microscope (Carl Zeiss Co., Ltd., Tokyo, Japan).

### 2.8. Bioinformatics and Data Analysis

Correlations between the expression level of IRF1 and that of signal transducer and activator of transcription 1 (STAT1), STAT2, STAT3 of HNSCC, and LUAD samples were obtained from TCGA cohorts through cBioportal for Cancer Genomics (https://www.cbioportal.org/, accessed on 19 May 2021).

### 2.9. Statistical Analysis

Data are presented as the mean ± standard deviation (SD) of at least 3 independent experiments. All statistical analyses were performed using GraphPad Prism software, version 9.01 (GraphPad Software, Inc., San Diego, CA, USA). A probability (*p*) value of < 0.05 was considered statistically significant.

## 3. Results

### 3.1. KPNB1 Regulates Cell Surface PD-L1 Expression of X-Ray-Irradiated A549 Cells

**Figure 1:**
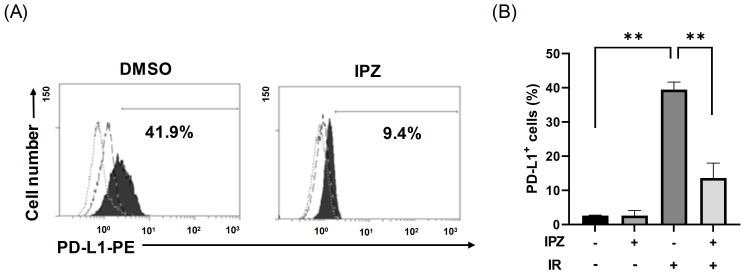
Effects of IPZ on radiation-increased PD-L1 expression on the surface of A549 cells. (**A**,**B**) IPZ (10 μM) was added to the culture medium at 1 h before X-ray irradiation at 6 Gy. After 4 days of culture, the cells were harvested for the analysis of cell-surface PD-L1 expression. (**A**) Representative histograms of PD-L1 expression on A549 cells are shown. The dotted and broken line histograms indicate isotype control and PD-L1 expression of non-irradiated cells, respectively. The filled black histogram indicates the PD-L1 expression of irradiated cells. Inset numbers indicate the percentage of PD-L1^+^ cells of 6 Gy-irradiated cells compared with an isotype control. (**B**) Data are presented as the mean ± SD of independent experiments. ** *p* < 0.01.

First, we investigated whether KPNB1 is involved in PD-L1 expression on the surface of irradiated LUAD (A549) cells. Since lung cancer is often treated with radiation therapy and our previous report demonstrated the involvement of KPNB1 in the malignancy of human LUAD cells [13], LUAD A549 cells were also used in the present study. In line with the findings of a previous report [7], X-ray irradiation increased PD-L1 expression on the surface of A549 cells (Figure 1A). As shown in Figure 1B, PD-L1 expression on the surface of X-ray-irradiated cells was significantly decreased by the KPNB1 inhibitor IPZ, but it had no effect on non-irradiated cells. These results indicate that KPNB1 regulates radiation-increased PD-L1 expression on the surface of A549 cells.

### 3.2. IRF1 Is Involved in Radiation-Increased PD-L1 Expression

**Figure 2:**
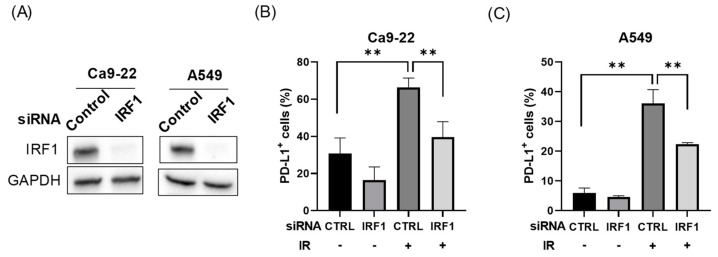
Involvement of IRF1 in radiation-increased PD-L1 expression in Ca9-22 and A549 cells. (**A**) Ca9-22 and A549 cells transfected with control or IRF1 siRNA were harvested for western blot analysis. An image of a representative immunoblot is shown. GAPDH was used as a loading control. (**B**,**C**) IRF1-knockdown Ca9-22 (**B**) and A549 cells (**C**) were irradiated with 6 Gy and cultured for 4 days. After culturing, the cells were harvested for analysis of cell surface PD-L1 expression. ** *p* < 0.01.

We next investigated whether IRF1 is involved in radiation-increased PD-L1 expression on the surface of IRF1-knockdown Ca9-22 and A549 cells (Figure 2A). Although IRF1 knockdown did not cause a significant decrease in PD-L1 expression under non-irradiated conditions, radiation-increased PD-L1 expression was significantly attenuated on the surface of both Ca9-22 and A549 cells (Figure 2B,C).

### 3.3. KPNB1 Is Involved in Nuclear Localization and Radiation-Increased Expression of IRF1

**Figure 3:**
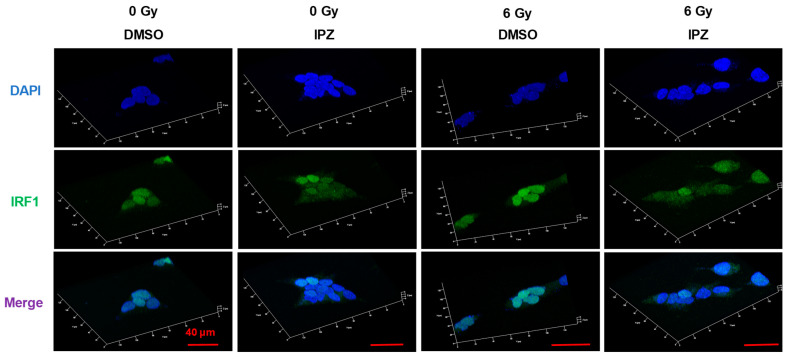
Effects of IPZ on the localization of IRF1 in Ca9-22 cells. IPZ (10 μM) was added to the culture medium at 1 h before irradiation at 6 Gy. After 24 h of culture, the treated Ca9-22 cells were harvested for the analysis of confocal images of IRF1. A 3D constructed image with maximum intensity is shown. Scale bar, 40 μm.

Next, the relationships of KPNB1 and IRF1 with radiation-increased PD-L1 expression were explored. First, the effect of IPZ on the nuclear localization of IRF1 in Ca9-22 cells was investigated by immunofluorescence analysis. As shown in Figure 3, nuclear localization of IRF1 was observed in DMSO-treated cells, while IPZ treatment resulted in less nuclear localization of IRF under both irradiated and non-irradiated conditions, indicating that KPNB1 is involved in the translocation of IRF1 into the nuclei of Ca9-22 cells.

**Figure 4:**
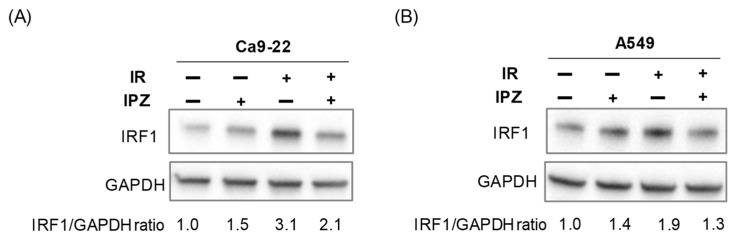
Effects of IPZ on IRF1 expression in irradiated Ca9-22 and A549 cells. (**A**,**B**) IPZ (10 μM) was added to the culture medium 1 h before irradiation at 6 Gy. The Ca9-22 (**A**) and A549 cells (**B**) were cultured for 24 and 48 h, respectively, and then harvested for western blot analysis. An image of a representative immunoblot is shown. GAPDH was used as a loading control.

Intriguingly, IRF1 knockdown did not decrease PD-L1 expression in non-irradiated cells (Figure 2B,C). DSBs induced by ionizing radiation and chemotherapeutic agents reportedly increase IRF1 expression, which is required for the upregulation of PD-L1 expression [7]. Therefore, the potential involvement of KPNB1 in radiation-increased IRF1 expression was investigated. As shown in Figure 4, upregulation of IRF1 expression in X-ray-irradiated cells was attenuated by IPZ treatment. Taken together, these results indicate that KPNB1 is involved in not only nuclear localization of IRF1 but also radiation-increased IRF1 expression.

### 3.4. KPNA2 Mediates Radiation-Increased PD-L1 Expression Through Nuclear Localization of IRF1

**Figure 5:**
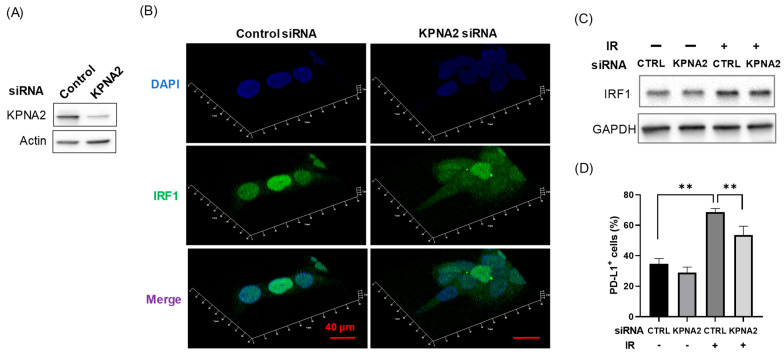
Effects of KPNA2 knockdown on radiation-increased IRF1 and PD-L1 expression in Ca9-22 cells. (**A**,**B**) Ca9-22 cells transfected with control or IRF1 siRNA were harvested for western blot analysis of confocal images of IRF1. (**A**) An image of a representative immunoblot is shown. Actin was used as a loading control. (**B**) Confocal images of IRF1 in Ca9-22 cells are shown. The images are 3D constructions with maximum intensity. Scale bar, 40 μm. (**C**) IRF1-knockdown Ca9-22 cells were irradiated with 6 Gy and cultured for 24 h. Afterward, the cells were harvested for western blot analysis of IRF1 expression. An image of a representative immunoblot is shown. GAPDH was used as a loading control. (**D**) IRF1-knockdown Ca9-22 cells were irradiated with 6 Gy and cultured for 4 days. Afterward, the cells were harvested for analysis of cell surface PD-L1 expression. ** *p* < 0.01. CTRL, control.

The above results suggest that KPNB1 mediates radiation-increased PD-L1 expression through upregulation and/or nuclear localization of IRF1. However, it is not clear whether nuclear localization of IRF1 is involved in radiation-increased PD-L1 expression. Therefore, the importance of nuclear localization of IRF in radiation-increased PD-L1 expression was investigated by focusing on KPNA2, which carries IRF1 into the nucleus [16]. Unsurprisingly, KPNA2-knockdown Ca9-22 cells showed less nuclear localization of IRF (Figure 5A,B). Importantly, although KPNA2 knockdown had a limited effect on radiation-increased IRF1 expression (Figure 5C), the radiation-increased PD-L1 expression of Ca9-22 cells was significantly attenuated (Figure 5D).

## 4. Discussion

PD-L1 is an immune checkpoint molecule that negatively regulates anti-tumor immunity and is associated with the clinical outcomes of cancer patients [3,4,5]. Although radiation therapy is an effective treatment for HNSCC and lung cancer, ionizing radiation is known to increase PD-L1 expression on the surface of cancer cells [6,7]. Hence, combination treatments of anti-PD-L1/PD-1 antibodies and radiation therapy have been tested in preclinical and clinical studies [19,20,21]. However, only a minority of patients have achieved durable responses after treatment with anti-PD-1/PD-L1 antibodies [22,23], suggesting that predictive markers are needed to optimize patient selection for the application of anti-PD-1/PD-L1 antibody therapies [24,25]. Therefore, another approach to overcome radiation-increased PD-L1 expression is needed. We previously reported that KPNB1 inhibition resulted in not only radiosensitization but also attenuation of radiation-increased PD-L1 expression in HNSCC cells [13], indicating that co-treatment with KPNB1 blockage and ionizing radiation is a promising strategy for HNSCC therapy. However, the mechanism underlying KPNB1-mediated, radiation-increased PD-L1 expression remains unknown. The results of the present study showed that KPNB1 inhibitor IPZ reduces radiation-increased PD-L1 expression by modulating the expression and nuclear import of IRF1.

In this study, IPZ attenuated radiation-increased PD-L1 expression on the surface of A549 cells without affecting the PD-L1 expression of non-irradiated cells (Figure 1). We previously observed similar effects in HNSCC cells [13], suggesting that KPNB1 is specifically involved in radiation-increased PD-L1 expression. Sato et al. reported that radiation increased IRF1 expression and that IRF1 knockdown attenuated radiation-increased PD-L1 expression in osteosarcoma U2OS and lung cancer H1299 cells [7]. In line with their report, similar results were observed in this study using HNSCC Ca9-22 and A549 cells (Figure 2 and Figure 4). Intriguingly, the results of the present study and previous research by Sato et al. [7] showed that IRF1 knockdown had only a limited effect on PD-L1 expression of non-irradiated cells (Figure 2). Therefore, IRF1 upregulation after irradiation might be required for radiation-increased PD-L1 expression. Thus, the limited effect of IPZ on PD-L1 expression of non-irradiated cells could be due to insufficient IRF1 expression to induce PD-L1 expression under non-irradiated conditions.

The main role of members of the karyopherin family is to carry karyophilic proteins with NLSs into the nucleus. The present results showed that both KPNB1 inhibitor and KPNA2 knockdown resulted in less nuclear localization of IRF1 (Figure 3 and Figure 5B). Liang et al. reported that KPNB1 and KPNA2 are eventually formed the complex with cargo proteins in the cytoplasm and translocated into the nucleus [26]. Therefore, it is thought that KPNB1 and KPNA2 synergistically transport IRF1 into the nucleus.

Here, we found that IPZ treatment resulted in not only less nuclear localization of IRF1 but also a decrease in IRF1 upregulation after irradiation (Figure 3 and Figure 4), suggesting that KPNB1 mediates radiation-increased PD-L1 expression via upregulation and/or nuclear localization of IRF1 (Appendix A and Figure S1). Fortunately, KPNA2 knockdown caused less nuclear localization of IRF1 and a significant decrease in radiation-increased PD-L1 expression without attenuating IRF1 upregulation after irradiation (Figure 5). Taken together, these results suggest that nuclear import of IRF1 is involved in radiation-increased, KPNB1-mediated PD-L1 expression. In addition, less nuclear localization of IRF1 by IPZ and KPNA2 knockdown failed to decrease PD-L1 expression of non-irradiated cells, which highlights the importance of IRF1 upregulation for radiation-increased PD-L1 expression.

The present study showed that not KPNA2 knockdown but KPNB1 inhibitor reduced radiation-increased IRF1 expression. It is reported that the activation of Janus kinases-STAT1/STAT2/STAT3 axis by interferon-γ stimulation increases IRF1 expression leading to the upregulation of PD-L1 [17]. Our in silico analysis using TCGA cohort revealed correlations between IRF1 and STAT1 or STAT2 mRNA in HNSCC and LUAD (Figure S2), suggesting that STAT1 and/or STAT2 may be mainly involved in PD-L1 expression in these cancers. Since KPNA1, not KPNA2, is known to transport STAT1 and STAT2 into the nucleus [27,28], there is a possibility that the KPNB1 inhibitor reduced radiation-increased IRF1 expression by inhibiting nuclear import of the KPNA1/STAT1/STAT2 complex. Therefore, it would be interesting to investigate the role of STAT1 and STAT2 as well as KPNA1 in radiation-increased KPNB1-mediated IRF1 expression in these cancers.

We previously reported that KPNB1 inhibition reduced radiation-increased PD-L1 expression and enhanced radiation-induced cell death in HNSCC cells [13]. However, there was the issue that KPNB1 inhibition exerts cytotoxicity against not only cancer cells but also normal cells [13]. This may be because KPNB1 inhibition affects the transport of all KPNA subtypes. The present study showed that KPNA2 inhibition reduced radiation-induced PD-L1 expression. Since KPNA2 selectively recognizes IRF1 compared with KPNB1, KPNA2 inhibition may overcome the issue. In addition, there are reports that KPNA2 is overexpressed in various cancers including lung cancer cells and is related to cancer malignancies [12,29]. Furthermore, Song et al. reported that the inhibition of KPNA2 augments radiation-induced cell death in colon cancer and breast cancer cells [30]. Therefore, although further studies investigating the cytotoxic effect of KPNA2 inhibition and the role of KPNA2 in radiation response in LUAD and HNSCC cells are needed, KPNA2 may be a potential target to improve radiation therapy.

## 5. Conclusions

The results of this study demonstrate that upregulation of IRF1 after irradiation is required for radiation-increased PD-L1 expression and that KPNB1 mediates radiation-increased, cell-surface PD-L1 expression through both upregulation and nuclear import of IRF1 in HNSCC and LUAD cells.

## Data Availability

The data presented in this study are available in the article.

## Supplementary Materials

The following are available online at https://www.mdpi.com/article/10.3390/cimb43010013/s1, Figure S1: Increases in cell surface PD-L1 expression in Ca9-22 cells at 24 h and 48 h after irradiation. Figure S2: Correlation between IRF1 and STAT1 or STAT3 mRNA expression in HNSCC and LUAD from TCGA.

## Appendix A

In the present study, we analyzed the expression and localization of IRF1 24 h after irradiation in Ca9-22 cells (Figure 3 and Figure 4), whereas the cell surface PD-L1 expression was analyzed 4 days after irradiation (Figure 2). This may make the reader think that there is a big gap between the changes in IRF1 and the upregulation of PD-L1 expression. However, as shown in Figure S1, the increase in cell surface PD-L1 expression occurred 24 h and 48 h after irradiation in Ca9-22 cells. Therefore, we consider that the changes in IRF1 observed at 24 h after irradiation is related to the radiation-increased cell surface PD-L1 expression in Ca9-22 cells.
