# Supplementary material for: KPNB1 Inhibitor Importazole Reduces Ionizing Radiation-Increased Cell Surface PD-L1 Expression by Modulating Expression and Nuclear Import of IRF1
Source: Curr Issues Mol Biol. 2021 May 19;43(1):153–62. doi: 10.3390/cimb43010013 (PMC8929148; doi:10.3390/cimb43010013)
Supplement: Supplementary file 1 [file cimb-43-00013-s001.zip › cimb-1212351-supplementary.pdf]

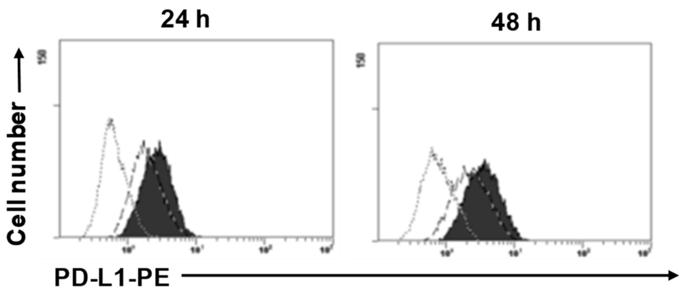


**Figure S1.** Increases in cell surface PD-L1 expression in Ca9-22 cells at 24 h and 48 h after irradiation.

Ca9-22 cells exposed to 6 Gy X-ray irradiation were cultured for 24 h and 48 h. The cells were harvested for analysis of cell surface PD-L1 expression. Representative histograms of PD-L1 expression are shown. The dotted line and broken line histograms indicate isotype control and PD-L1 expression of non-irradiated cells, respectively. The filled black histogram indicates PD-L1 expression of irradiated cells.

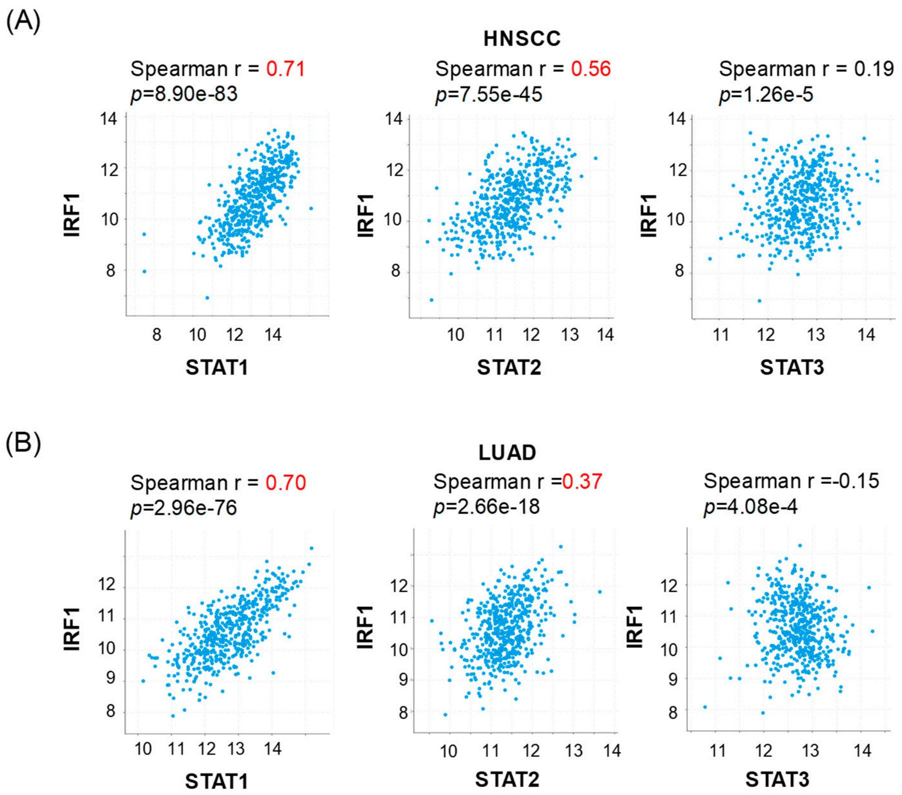


**Figure S2.** Correlation between IRF1 and STAT1 or STAT3 mRNA expression in HNSCC and LUAD from TCGA.

(**A**, **B**) Correlations between IRF1 and STAT1, STAT2, or STAT3 mRNA expression in HNSCC and LUAD cohorts from TCGA were analyzed through cBioportal for Cancer Genomics. (**A**) TCGA HNSCC Firehose Legacy 528 patients / 530 samples, (**B**) TCGA LUAD Firehose Legacy 584 patients / 586 samples.
